# Supplementary material for: Bone Morphogenetic Protein 2 (BMP-2) Aggregates Can be Solubilized by Albumin—Investigation of BMP-2 Aggregation by Light Scattering and Electrophoresis
Source: Pharmaceutics. 2020 Nov 25;12(12):1143. doi: 10.3390/pharmaceutics12121143 (PMC7760923; doi:10.3390/pharmaceutics12121143)
Supplement: Supplementary file 1 [file pharmaceutics-12-01143-s001.pdf]

pharmaceutics

MDPI

Supplementary Materials: Bone Morphogenetic Protein 2 (BMP-2) Aggregates Can Be Solubilized by Albumin – Investigation of BMP-2 Aggregation by Light Scattering and Electrophoresis

Julius Sundermann, Holger Zagst, Judith Kuntsche, Hermann Wätzig and Heike Bunjes *

1. icIEF Measurements Using Urea

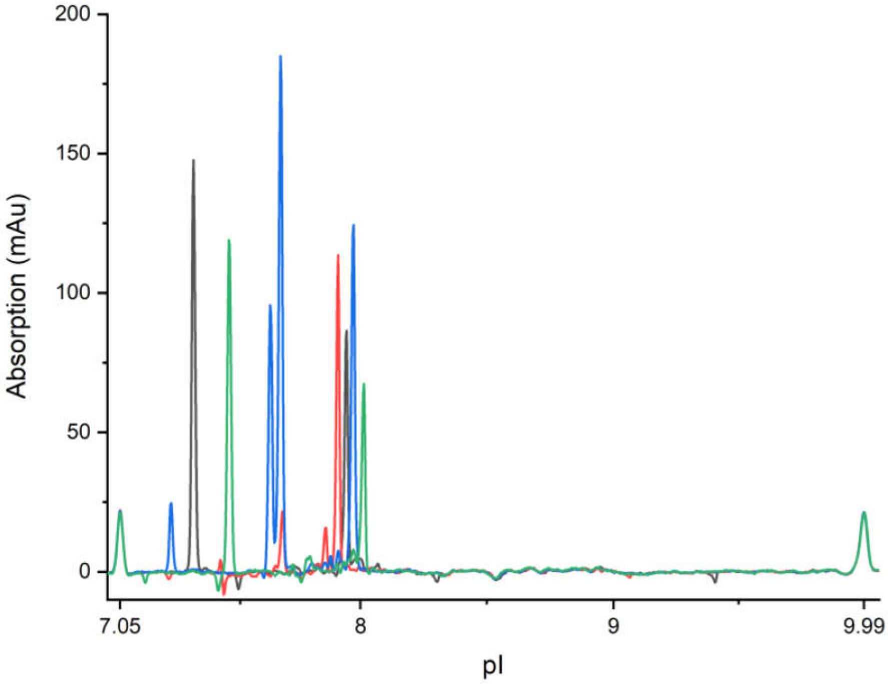

Figure S1. Superimposed electropherograms of four consecutive runs of a BMP-2 sample solution consisting of: 39 µL 1% methyl cellulose, 44 µL 10 M urea, 2.2 µL 500 mM L-arginine, 4.4 µL Pharmalyte 3-10, 0.55 µL pI marker 7.05 (1 mg/mL), 0.55 µL pI marker 9.99 (1 mg/mL), 14.4 µL water, 5.5 µL BMP-2 (1 mg/mL in 1 mM HCl); other method parameters as described in 2.1 except that the detection was done by UV absorbance at 280 nm.

2. DLS Measurements

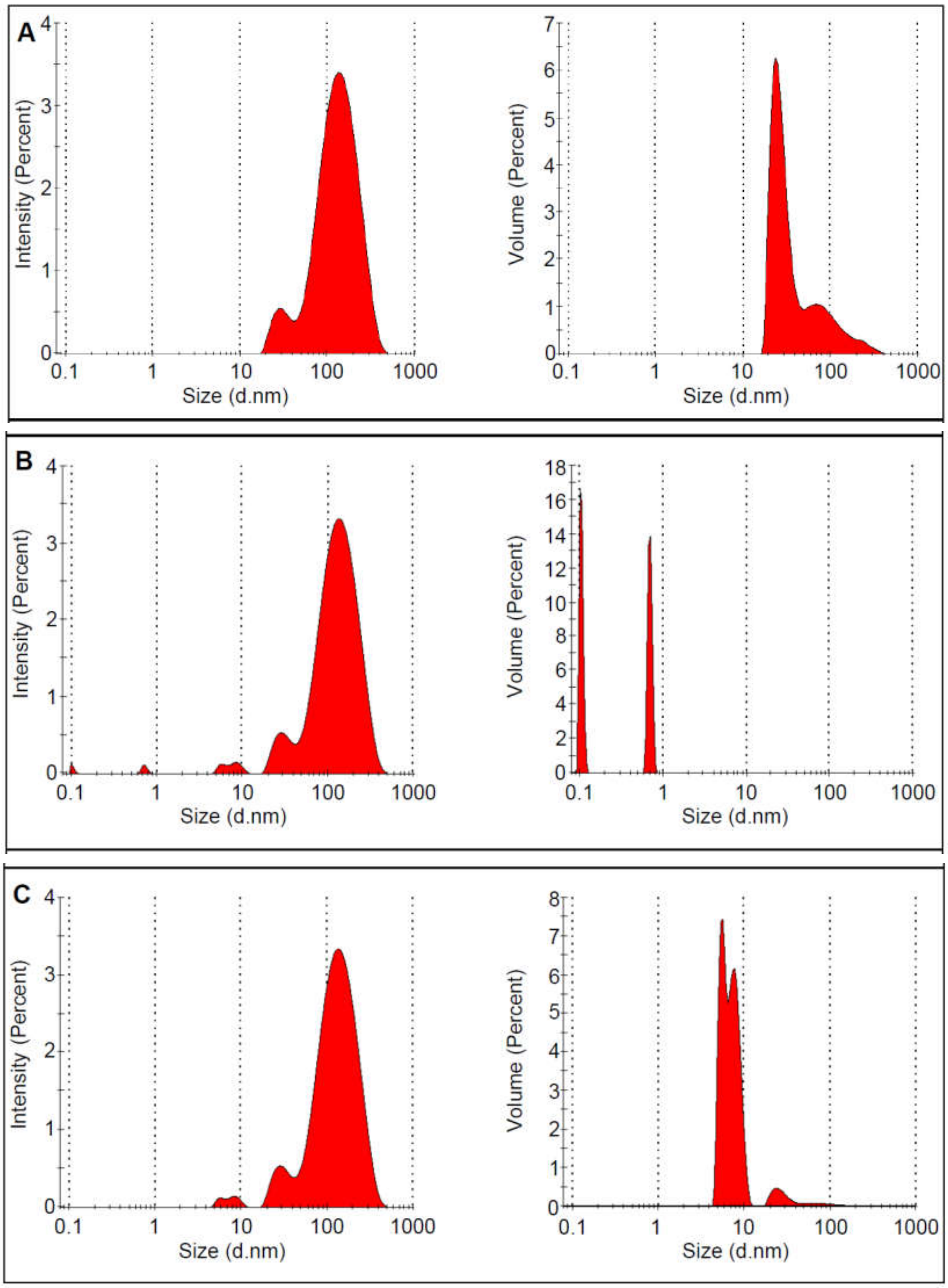

Figure S2. DLS-based size distribution of a representative measurement of BMP-2 in MES buffer pH 5 by intensity (left) and volume (right). Panel shows three results based on the same data set: (**A**) default settings, lower threshold: 0.05, lower limit: 0.01 nm (**B**) lower threshold: 0.00, lower limit: 0.01 nm (**C**) lower threshold: 0.00, lower limit: 1 nm.

3. Plot of *E. coli* BMP-2 IEF Results from Uludag et al.

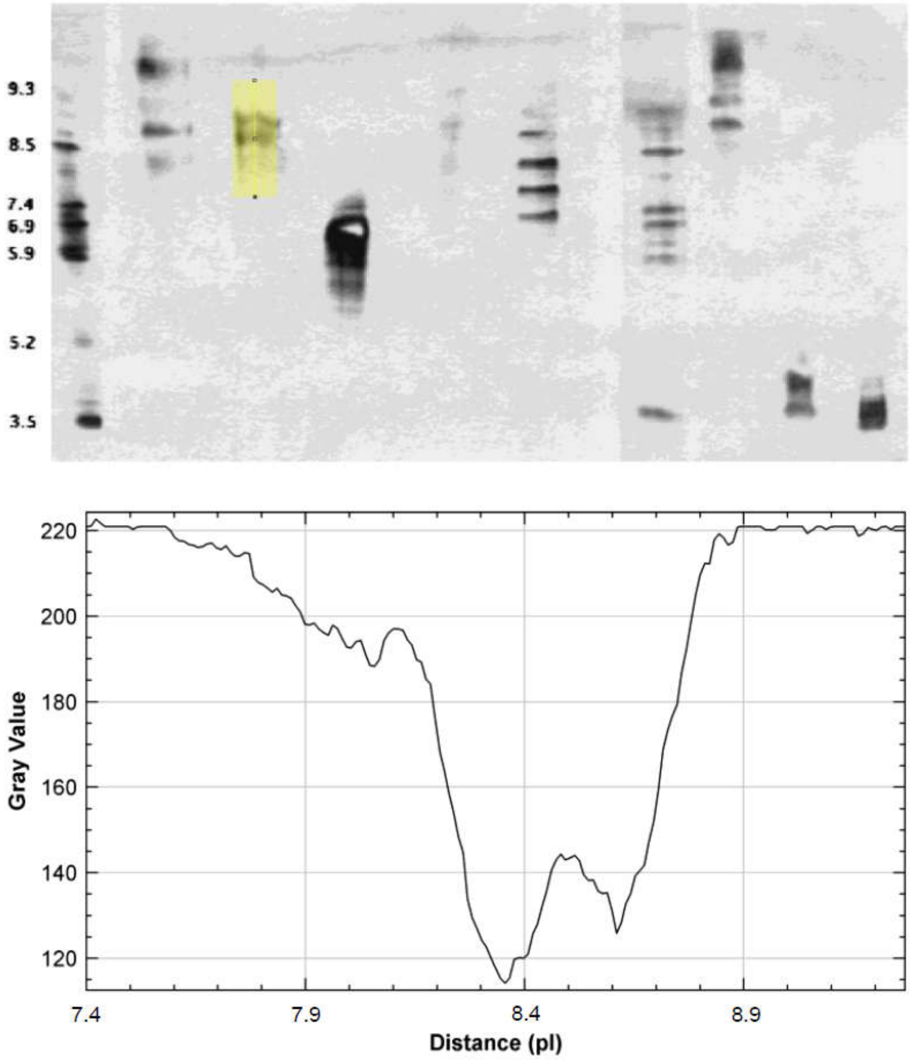

Figure S3. Top: IEF gel from Uludag, D'Agusta et al. [1]. Original figure caption: "A representative gel for determination of the rhBMP pIs. The shown pI markers (Lanes 1 and 7 from left) were from Bio-Rad. Lanes 2 and 8 were CHO rhBMP-2; Lane 3 was *E. coli* rhBMP-2; Lane 4 was plasmin cleaved rhBMP-2; Lane 5 was rhBMP-6; Lane 6 was rhBMP-4; Lane 9 was acetylated rhBMP-2; and Lane 10 was succinylated rhBMP-2." The yellow marked area on top of the E. coli BMP-2 lane is not part of the original figure. It marks the area within which the average gray value was plotted (bottom graph) along the yellow line using the Fiji software [2]. Based on the displayed scale, the examined pI range was estimated to be 7.4–9.4. Bottom: corresponding plot.

References

1. Uludag, H.; D'Augusta, D.; Golden, J.; Timony, G.; Li, J.; Riedel, R.; Wozney, J.M. Implantation of recombinant human bone morphogenetic proteins with biomaterial carriers: A correlation between protein pharmacokinetics and osteoinduction in the rat ectopic model. *J. Biomed. Mater. Res.* **2000,** *50* 227–238.
2. Schindelin, J.; Arganda-Carreras, I.; Frise, E.; Kaynig, V.; Longair, M.; Pietzsch, T.; Preibisch, S.; Rueden, C.; Saalfeld, S.; Schmid, B.; Tinevez, J.-Y.; et al. Fiji: an open-source platform for biological image analysis. *Nat. Methods* **2012**, *9*, 676–682, doi:10.1038/nmeth.2019 7.47.98.48.9
